# Features Split and Aggregation Network for Camouflaged Object Detection

**DOI:** 10.3390/jimaging10010024

**Published:** 2024-01-18

**Authors:** Zejin Zhang, Tao Wang, Jian Wang, Yao Sun

**Affiliations:** 1 HDU-ITMO Joint Institute, Hangzhou Dianzi University, Hangzhou 310018, China; zhangzejin@hdu.edu.cn (Z.Z.); wangtao21@hdu.edu.cn (T.W.); sunyao@hdu.edu.cn (Y.S.); 2 School of Automation, Hangzhou Dianzi University, Hangzhou 310018, China

**Keywords:** bio-inspired network, context-aware features, multi-scale features, camouflaged object detection

## Abstract

Higher standards have been proposed for detection systems since camouflaged objects are not distinct enough, making it possible to ignore the difference between their background and foreground. In this paper, we present a new framework for Camouflaged Object Detection (COD) named FSANet, which consists mainly of three operations: spatial detail mining (SDM), cross-scale feature combination (CFC), and hierarchical feature aggregation decoder (HFAD). The framework simulates the three-stage detection process of the human visual mechanism when observing a camouflaged scene. Specifically, we have extracted five feature layers using the backbone and divided them into two parts with the second layer as the boundary. The SDM module simulates the human cursory inspection of the camouflaged objects to gather spatial details (such as edge, texture, etc.) and fuses the features to create a cursory impression. The CFC module is used to observe high-level features from various viewing angles and extracts the same features by thoroughly filtering features of various levels. We also design side-join multiplication in the CFC module to avoid detail distortion and use feature element-wise multiplication to filter out noise. Finally, we construct an HFAD module to deeply mine effective features from these two stages, direct the fusion of low-level features using high-level semantic knowledge, and improve the camouflage map using hierarchical cascade technology. Compared to the nineteen deep-learning-based methods in terms of seven widely used metrics, our proposed framework has clear advantages on four public COD datasets, demonstrating the effectiveness and superiority of our model.

## 1. Introduction

**Figure 1:**
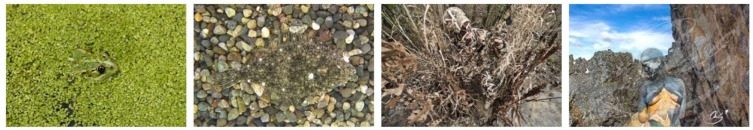
Examples of camouflaged objects; from left to right are natural camouflaged objects and artificial camouflaged objects.

When viewing an image or encountering a scene, it can be challenging to notice the object at first glance if the difference between the foreground and background is minimal [1,2,3,4,5,6]. The attempt by one or more objects to modify their traits (such as color, texture, etc.) to blend into their environment to avoid discovery is known as camouflage. There are two types of camouflage, as depicted in Figure 1. Natural camouflaged objects [7,8] are animals that use their inherent advantages to blend in with their surroundings and protect themselves. For instance, chameleons or other animals can change their color and physical appearance to match the hues and patterns of their surroundings. Additionally, artificial camouflaged objects were initially employed in battle, where soldiers and military gear employ camouflage to blend into their surroundings. In daily life, we can also see artificial camouflage, such as body art. Because of the characteristics of the camouflaged objects, the study of COD has not only scientific value but also significant engineering applications, such as surface defect detection [9], polyp segmentation [10], pest control, search, and rescue [11,12], and other applications.

The research on camouflage can be traced back to 1998. In recent years, COD has attracted more and more attention from researchers. Traditional models are mainly based on hand-crafted features (color, texture, optical flow, etc.) to describe the unified features of the object [13,14,15,16]. However, limited by hand-crafted features, the traditional model cannot work well when the background environment changes [17,18]. To address this, deep-learning-based techniques for COD have been developed, which utilize deep features automatically learned by the network from extensive training images. These features are more generic and effective than hand-crafted features. For example, Fan et al. [19] designed the first deep-learning-based model, SINet, which simulates the human visual mechanism, especially used for COD. Zheng et al. [20] successfully predicted the camouflage map using the short connection of the frame. However, there are still some shortcomings in the existing models. Specifically, (1) they cannot deeply explore high-level features, leading to imprecisely locating small objects. (2) There is no particularly effective method for integrating high-level and low-level features, even directly discarding low-level features, resulting in suboptimal performance in handling object edges.

**Figure 2:**
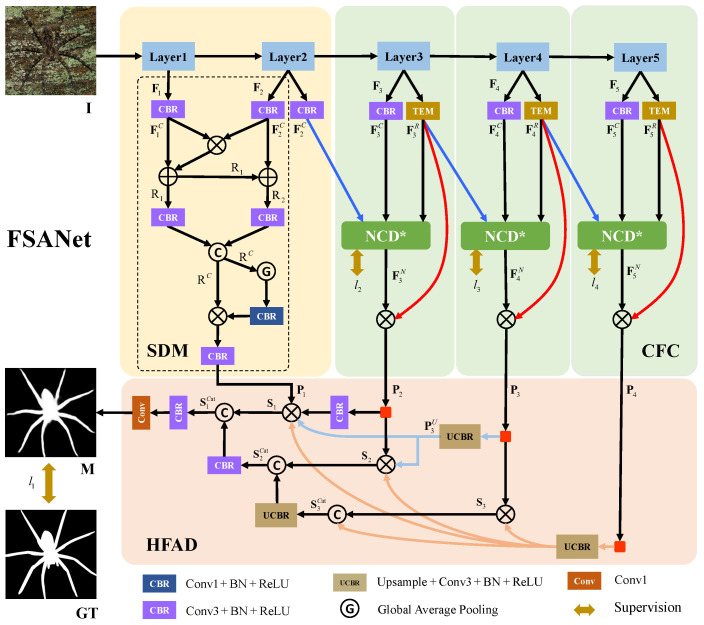
The overall architecture of the proposed FSANet, which can be divided into three key components; they are spatial detail mining module, cross-scale feature combination module, and hierarchical feature aggregation decoder. The input is camouflaged object I , and the result is prediction map M .

Inspired by the description above, we propose a new model for COD named features split and aggregation network (FSANet) to solve the above problems, as shown in Figure 2, which primarily consists of three modules. Taking into account how low-level features and high-level features contribute differently to the creation of camouflage maps [19,20], we use the backbone to extract five feature layers and divide them into two parts with the second layer as the boundary. The first stage consists of the backbone’s first two layers, simulating a human’s cursory examination of the scene to gather spatial details (such as the edge, texture, etc.). The SDM module is used in this phase to fuse the features to create a cursory impression. The second stage consists of the backbone’s last three layers, simulating a person’s additional observation and reworking of imperfect scenes. Specifically, both the same and different information is observed for the same feature across different viewing angles. We employ an ordinary convolution layer and TEM [1] module to mimic the human evaluation of features from various angles. After that, we fuse these features to enhance similar features. To avoid detail distortion and filter out noise by feature multiplication, we simultaneously utilize side-join multiplication (SJM), as shown by the red line in Figure 2. These operations constitute the CFC module. Finally, to obtain more thorough detection results, we build the HFAD module to thoroughly mine effective information from the two stages. We guide the fusion of low-level features by using high-level semantic information, and we enhance the camouflage map generated in the earlier stage by using a hierarchical cascade technique.

Overall, we can summarize our main contributions as follows:

1. We simulate the human observation camouflage scenes to propose a new COD method that includes the spatial detail mining module, the cross-scale feature combination module, and the hierarchical feature aggregation decoder. We rigorously test our model against nineteen others using four public datasets (CAMO [21], CHAMELEON [22], COD10K [1], and NC4K [2]) and evaluate it across seven metrics, where it demonstrates clear advantages.
2. To fully mine spatial detail information, we design a spatial detail mining module that interacts with first-level feature information, simulating the human’s cursory examination. To effectively mine information in high-level features, we designed a cross-scale feature combination module to strengthen high-level semantic information by combining features from adjacent scales, simulating humans’ evaluation of features from various angles. Furthermore, we build a hierarchical feature aggregation module to fully integrate multi-level deep features, simulating humans’ aggregation and processing of information.

## 2. Related Works

This section discusses COD based on deep learning and context-aware deep learning, both of which are related to our model.

### 2.1. Camouflaged Object Detection (COD)

Camouflaged Object Detection (COD) has become an important area of research for identifying objects that are blended in with their surroundings. In this emerging field, significant contributions have been made.

Fan et al. [19] utilized a search module (SM) alongside a partial decoder component (PDC) [23] to enhance the accuracy of initial detection zones, fine-tuning the identification of camouflaged objects by focusing on salient features within the rough areas. Sun et al. [24] developed C^2^F-Net, which employs multi-scale channel attention to guide the fusion of features across different levels. Their approach ensures that both local nuances and global context are considered, thus improving the detection of objects across various scales. Mei et al. [3] introduced PFNet, which cleverly combines high-level feature maps with inverted predictions. By integrating these with the current layer’s attributes, and processing them through a context exploration block, the network is able to effectively reduce false positive and negative detections by strategically employing subtraction techniques. Li et al. [25] proposed JCOSOD, which considers the uncertainties inherent in fully labeling camouflaged objects. They used a full convolutional discriminator to gauge the confidence in predictions, and an adversarial training strategy was applied to refine the model’s ability to estimate prediction confidence.

Together, these advancements reflect a growing sophistication in COD, showing a trend towards more nuanced algorithms capable of distinguishing objects that are naturally or artificially designed to be hard to detect.

### 2.2. Context-Aware Deep Learning

Contextual information is important in object segmentation tasks as it has the ability to improve feature representation and, in turn, improve performance. Efforts have been made to improve contextual information. Stars et al. [26] proposed a salience detection algorithm based on four psychological principles. The model defines the algorithm using local low-level considerations, global considerations, and visual organization rules. High-level factors are used for post-processing and are helpful in producing compact, attractive, and rich information. Chen et al. [27] created ASPP, which collects contextual data using various dilated convolutions. They proposed an approach that, in the end, produces accurate semantic segmentation results based on the DCNN’s capability to detect objects and the fcCRF’s capability to localize objects with fine detail. To improve the features of the local context, Tan et al. [28] employed LCANet to merge the local area context and the global scene context in a coarse-to-fine framework.

## 3. The Proposed Method

In this section, we present the overall architecture of FSANet before delving into the specifics of each module. Finally, we discuss the training loss function of the proposed model.

### 3.1. Overall Architecture

The overall architecture of FSANet can be seen in Figure 2, which consists primarily of the spatial detail mining module, the cross-scale feature combination module, and the hierarchical feature aggregation decoder to endow the model with the ability to detect camouflaged objects. Specifically, for the input image $I\in R^{W\times H\times 3}$ , we use Res2Net-50 [29] as the backbone to extract five different levels of information, denoted as $F_{i},i\in \left\{1,2,3,4,5\right\}$ . The resolutions of each layer are $\left\{\left[\frac{H}{k},\frac{W}{k}\right],k=4,4,8,16,32\right\}$ . We divide the backbone into two parts, with the second layer as a boundary. The first two levels of features are low-level fine-detail features, including spatial details (such as edge, texture, etc.), while the last three layers $F_{i},i\in \left\{3,4,5\right\}$ are high-level semantic features that include specific details (such as semantic information, position, etc.). We obtain the spatial details from low-level features by using the spatial detail mining module and combine the features to provide a superficial impression, denoted by $P_{1}$ . However, it contains more redundant information. For high-level features, we design a cross-scale feature combination module to obtain three layers of high-level features denoted as $P_{i},i\in \left\{2,3,4\right\}$ , each with different specific semantic information. Finally, we employ the hierarchical feature aggregation decoder, which utilizes the high-level features layer to refine and fuse the low-level features layer by layer, yielding the prediction map of COD. Below are detailed descriptions of each key component.

### 3.2. Spatial Detail Mining (SDM)

Because the camouflaged object is very similar to the background, the extracted low-level features contain rich spatial detail and adjacent features have great similarity, but they also contain more noise information. Therefore, we use this module to find similar features and eliminate noise, simulating a human’s cursory examination of the scene to gather spatial details. To retain the fine-detail information in the low-level features while discarding the noise information and unsuitable features, we combine the adjacent features of the first two layers $F_{i}^{C},i\in \left\{1,2\right\}$ , with element-wise multiplication on these two features to extract shared features, followed by element-wise addition. We obtain $R_{1}$ and $R_{2}$ after these operactions. Then, we concatenate $R_{1}$ and $R_{2}$ to obtain $R^{C}$ . Global average pooling is applied to $R^{C}$ to weight the features, and these are further enhanced with local information through element-wise multiplication with the original feature $R^{C}$ . Finally, the channels of feature are reduced to 32 through convolution to obtain feature $P_{1}$ with the size $\left\{\left[\frac{H}{k},\frac{W}{k},32\right],k=4\right\}$ . The spatial detail mining process can be described as follows:

(1) $$\left\{\begin{array}{c} R_{1}=F_{1}^{C}⊗F_{2}^{C}⊕F_{1}^{C}\\ R_{2}=R_{1}⊕F_{2}^{C}\\ R^{C}=Concat\left\{CBR\left(R_{1}\right),CBR\left(R_{2}\right)\right\}\\ P_{1}=CBR\left(R^{C}⊗CBR\left(G\left(R^{C}\right)\right)\right) \end{array}\right.$$

where $CBR\left(\cdot \right)$ represents the Conv+BN+ReLU operation and $Concat\left\{\cdot \right\}$ represents concatenate operation in dim = 1, $G\left(\cdot \right)$ represents global average pooling operation, which is used to establish relationships between feature maps and categories.

### 3.3. Cross-Scale Feature Combination (CFC)

Different types of camouflaged objects have varying colors, physical traits, and camouflage techniques. Similarly, objects of the same type that are camouflaged can have different camouflage methods and sizes in various environments, making it more challenging to locate them. In studies on biological vision, researchers have been discussing challenges of perspective. Viewpoint-invariant theories and viewpoint-dependent theories have been proposed [30,31,32]. Viewpoint-invariant theories assert that a particular object can be recognized from diverse viewing angles while maintaining its properties. On the other hand, viewpoint-dependent theories suggest that object recognition from different viewing angles may be effective. Separately, Tarr et al. [33] proposed a multi-view model in which objects can be represented by a series of images of familiar viewpoints, with each view describing a different view-specific object characterization.

Inspired by this, we realize that using various receptive fields with reduced-channel operations can provide additional feature information about the objects. Thus, we design the cross-scale feature combination module, which processes features and adjacent features differently to obtain different viewpoints and finally fuses them to obtain advanced features, simulating the person’s additional observation and reworking of imperfect scenes.

Specifically, we utilize the Conv3 to handle the features $F_{i},i\in \left\{2,3,4,5\right\}$ to preserve object boundaries and enhance local context information, and we use the TEM [1] to handle the features $F_{i},i\in \left\{3,4,5\right\}$ to capture multi-scale information further. This enables us to obtain $F_{i}^{C},i\in \left\{2,3,4,5\right\}$ and $F_{i}^{R},i\in \left\{3,4,5\right\}$ . All features’ channels are adjusted to 32. After that, we use NCD*, which selective removes upsampling from NCD [1] to ensure dimensional consistency, fine-tune, and effectively combine features from different viewpoints; the inputs are $F_{i-1}^{C}$ , $F_{i}^{C}$ , and $F_{i}^{R}$ with output $F_{i}^{N}$ .

However, since camouflaged objects are relatively blurred, using NCD* may result in detail and other useful information loss while enhancing similar features. Therefore, we use side-join multiplication to re-add details to the output features and filter out noise through multiplication to further enhance the object’s features; obtain $P_{i},i\in \left\{3,4,5\right\}$ with the size $\left\{\left[\frac{H}{k},\frac{W}{k},32\right],k=4,4,8\right\}$ . This operation is depicted by the red line in Figure 2. The cross-scale feature combination process can be described as follows:

(2) $$\left\{\begin{array}{c} F_{i}^{N}=N\left\{\mathrm{CBR}\left(F_{i-1}\right),CBR\left(F_{i}\right),T\left(F_{i}\right)\right\}\;\;i=3\\ F_{i}^{N}=N\left\{R\left(F_{i-1}\right),CBR\left(F_{i}\right),T\left(F_{i}\right)\right\}\;\;\;\;\;i=4,5\\ P_{i-1}=F_{i}^{N}⊗R\left(F_{i}\right)\;\;\;\;\;\;\;i=3,4,5 \end{array}\right.$$

where $CBR\left(\cdot \right)$ represents Conv+BN+ReLU operation, $N\left\{\cdot \right\}$ represents neighbor connection decoder (NCD*), $T\left(\cdot \right)$ represents texture-enhanced module (TEM).

### 3.4. Hierarchical Feature Aggregation Decoder (HFAD)

We obtain improved features $\left\{P_{1},P_{2},P_{3},P_{4}\right\}$ using the method described above. The next crucial problem is how to successfully bridge the context and fuse these features. To address this, our model employs hierarchical cascade technology, which gradually guides the fusion of low-level features using high-level semantics, simulating human processing of aggregated information obtained from different sources. We regard the process of fusing rich features as a decoder. Formally, the hierarchical feature aggregation decoder contains four inputs, as shown in Figure 2. The module’s general structure is an inverted triangle hierarchical structure, primarily consisting of Conv3+BN+ReLU layers and element-wise multiplication to extract similarities between various features. To ensure that cascade processes may be completed, we resize the features to an appropriate size in the process by using an upsampling operation.

Specifically, we first apply an upsampling operation for $P_{4}$ to make it the same shape as $P_{3}$ , and we multiply them to obtain $S_{3}$ . Then, we upsample $P_{3}$ and $P_{4}$ , respectively, to match the size of $P_{2}$ , and multiply them to obtain $S_{2}$ . The same operations are applied to obtain $S_{1}$ . This progressive method is characterized as follows:

(3) $$\left\{\begin{array}{c} P_{3}^{U}=CBR\left(\delta_{↑}^{2}\left(P_{3}\right)\right)\\ S_{3}=CBR\left(\delta_{↑}^{2}\left(P_{4}\right)\right)⊗P_{3}\\ S_{2}=CBR\left(\delta_{↑}^{4}\left(P_{4}\right)\right)⊗P_{3}^{U}⊗P_{2}\\ S_{1}=CBR\left(\delta_{↑}^{4}\left(P_{4}\right)\right)⊗P_{3}^{U}⊗CBR\left(P_{2}\right)⊗P_{1} \end{array}\right.$$

where $CBR\left(\cdot \right)$ represents Conv+BN+ReLU operation. To ensure that the candidate features have the same size as each other, we use upsampling operation before element-wise multiplication; $\delta_{↑}^{2}\left(\cdot \right)$ means 2× upsampling operation by executing the bilinear interpolation, and $\delta_{↑}^{4}\left(\cdot \right)$ 4× upsampling operation.

After performing the above operation, we obtain three refined features, denoted by $\left\{S_{1},S_{2},S_{3}\right\}$ . Then, we use the concatenation operation with Conv3+BN+ReLU layers to enhance the feature step by step, obtaining $S_{3}^{Cat}$ , $S_{2}^{Cat}$ , and $S_{1}^{Cat}$ . Finally, we use a convolution layer to reduce the channels and obtain the final prediction map $M\in R^{W\times H\times 1}$ . The following formulas express this process:

(4) $$\left\{\begin{array}{c} S_{3}^{Cat}=Concat\left\{S_{3},CBR\left(\delta_{↑}^{2}\left(P_{4}\right)\right)\right\}\\ S_{2}^{Cat}=Concat\left\{S_{2},CBR\left(\delta_{↑}^{2}\left(S_{3}^{Cat}\right)\right)\right\}\\ S_{1}^{Cat}=Concat\left\{S_{1},CBR\left(S_{1}^{Cat}\right)\right\}\\ M=Conv\left(CBR\left(S_{1}^{Cat}\right)\right) \end{array}\right.$$

where $CBR\left(\cdot \right)$ represents Conv3+BN+ReLU operation. $\delta_{↑}^{2}\left(\cdot \right)$ means 2× upsampling operation by executing the bilinear interpolation. $Concat\left\{\cdot \right\}$ represents concatenating operation in dim = 1. Conv means 1×1 convolutional layer. Following these operations, we obtain the prediction map.

### 3.5. Loss Function

The binary cross-entropy (BCE) [34] loss, which highlights pixel-level differences, disregards discrepancies between neighboring pixels, and equally weights foreground and background pixels, is often employed in the binary classification problem. For object detection and segmentation, the IoU is a commonly used assessment metric that emphasizes global structure. Inspired by [35,36], we adopt weighted BCE loss and weighted IOU loss as the combined loss. Weighted BCE and weighted IoU losses place a greater focus on hard samples compared to regular BCE and IoU losses. The following formula shows how we define our loss:

(5) $$L=L_{IOU}^{w}+L_{BCE}^{w}$$

where $L_{IOU}^{w}$ and $L_{BCE}^{w}$ denote BCE loss and IoU loss, respectively. It has been proven successful to apply the same parameter definition and setup as [36,37].

The four supervision maps in this model are all closely supervised, and their locations are illustrated in Figure 2. Here, each map is enlarged through upsampling to align its dimensions with the GT. The total loss can be calculated using the formula below:

(6) $$L_{\mathrm{all}\;}=\sum_{i=1}^{4}L\left(l_{i},G\right)$$

The *G* represents the GT, and $L\left(l_{i},G\right)$ represent the loss calculation between each output and the ground truth, respectively.

## 4. Experimental Results

In this section, we will delve into greater detail about the benchmark datasets in the COD field, evaluation measures, experimental setup, and ablation study.

### 4.1. Datasets and Implementation

We conducted extensive comparisons on four publicly available COD datasets (CAMO [21], CHAMELEON [22], COD10K [1], and NC4K [2]) to fully validate our method.

**CAMO** [21] dataset includes 1250 photos and was suggested in 2019. It contains two types of scenes: indoor scenes (artworks) and outdoor scenes (disguised humans/animals). The dataset also includes a few images that are not camouflaged.

**CHAMELEON** [22] dataset includes 76 natural images, each of which is matched with an instance-level annotation. This dataset collection primarily focuses on creatures that are disguised in complicated backgrounds, making it challenging for humans to identify them from the environment.

**COD10K** [1] dataset includes 10K images, which are classified as 5066 camouflaged, 3000 background, and 1934 non-camouflaged images. The dataset is divided into five main categories and sixty-nine subcategories, including images of land, sea, air, and amphibians in camouflaged scenes as well as images of non-camouflaged environments.

**NC4K** [2] dataset includes 4121 images, which is the largest existing COD testing dataset. The dataset’s camouflaged scenes can be generally classified into two categories: natural camouflaged and artificial camouflaged, and the majority of the visual scenes in this collection are also naturally hidden.

**Implementation Details:** In this instance, we train our model using the same training dataset as stated in [1], which consists of 4040 images from the COD10K and CAMO datasets. The remaining images are used as testing datasets. Additionally, the training dataset is strengthened by randomly flipping images to increase the sufficiency of the network training, and each training image’s size is changed to 352×352 in the training phase. Our model is built with PyTorch and run on a PC with an NVIDIA GTX 2080Ti GPU. Parts of the parameters are initialized with Res2Net-50 [29] during the training process, while the remaining parameters are randomly initialized. The network is optimized using the Adam algorithm [38], with the initial learning rate, batch size, and maximum epoch number set to $10^{-4}$ , 16, and 100.

### 4.2. Evaluation Metrics

We use seven common metrics to evaluate and conduct a quantitative comparison of different models on COD datasets, including precision–recall ( PR ) curve, S-measure [39] ( $S_{m}$ ), F-measure [40] ( $F_{\beta }$ ), weighted F-measure [41] ( $F_{\beta }^{w}$ ), E-measure [42] ( $E_{m}$ ), and mean absolute error ( MAE ). Please refer to the evaluation code for details in https://github.com/DengPingFan/CODToolbox (accessed on 16 January 2024).

**Precision and recall** are common metrics used to evaluate how well the model works. The recall value is used as the horizontal coordinate and the precision value as the vertical coordinate to create a coordinate system. We can calculate the associated precision and recall scores to evaluate the effectiveness of the models.

**S-measure** [39] is used to determine the structural similarities between the prediction map and the related ground truth, which is defined as

(7) $$S=\alpha \times S_{o}+\left(1-\alpha \right)\times S_{r}$$

where $S_{o}$ represents the structural similarity measurement based on the object level and $S_{r}$ represents the region-based similarity. According to [39], the α is set to 0.5 .

**F-measure** [40] is used to calculate the weighted summation average of the precision and recall under non-negative weights. It is often used to compare the similarity of two images. The formula can be expressed as

(8) $$F_{\beta }=\left(1+\beta^{2}\right)\frac{PR}{\beta^{2}P+R}$$

where *P* represents precision and *R* represents recall. We set $\beta^{2}$ to 0.3, as suggested in [43], to emphasize precision. To improve the accuracy and completeness metrics, we determine the weights of recall and precision, as similarly conducted in [41]. The following is the formula:

(9) $$F_{\beta }^{w}=\left(1+\beta^{2}\right)\frac{P^{w}R^{w}}{\beta^{2}P^{w}+R^{w}}$$

The parameters are the same as $F_{\beta }$ , and *w* represents the weighted harmonic mean of the precision and recall.

**E-measure** [42] assesses the similarity between the prediction map and the ground truth by using the pixel significance value and the average significance value. The formula is as follows:

(10) $$\begin{array}{c} E=\frac{1}{W\times H}\sum_{x=1}^{W}\sum_{y=1}^{H}f\left(i\right) \end{array},$$

where f(·)=S(x,y)−G(x,y) stands for the enhanced alignment term, which is used to record statistics at the image level and pixel level. The image’s width and height are denoted by *W* and *H*.

**MAE** is used to quantify the average absolute difference between the model’s output and the input’s ground truth, which is the pixel-level error evaluation index. The formula can be written as follows:

(11) $$\mathrm{MAE}=\frac{1}{W\times H}\sum_{i=1}^{W\times H}\left|S(i)-G(i)\right|,$$

where S(i) represents the predicted map. G(i) represents the GT. *W* and *H* denote the image’s width and height.

### 4.3. Comparison with the State-of-the-Art Methods

In this part, we denote our model FSANet as “ours” and include some models from salient object detection and medical image segmentation to compare. We evaluate a total of ten models in salient object recognition and medical image segmentation, with nine models in COD, including EGNet [44], F^3^Net [37], SCRN [45], PoolNet [46], CSNet [47], SSAL [48], UCNet [49], MINet [50], ITSD [51], PraNet [10], PFNet [3], UJSC [25], SLSR [2], SINet [19], MGL-R [52], C^2^FNet [24], UGTR [53], SINet_V2 [1], and FAPNet [8]. The results of all these methods were obtained from publicly available data, created by the model, or retrained using the author’s code.

#### 4.3.1. **Quantitative Comparison**

**Figure 3:**
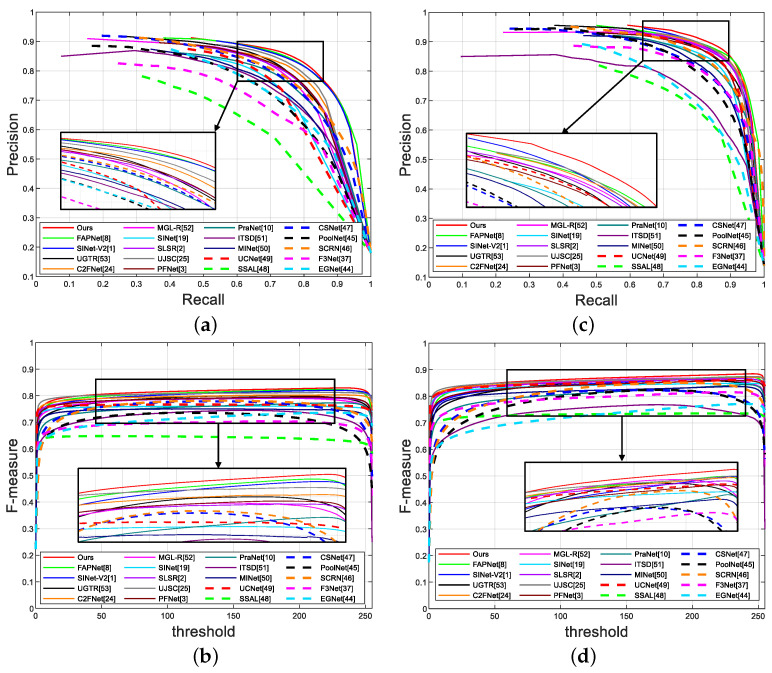
Quantitative evaluation of different models. The first row shows PR curves; the second row shows F-measure curves; (**a**,**b**) display the results for CAMO dataset; (**c**,**d**) display the results for CHAMELEON dataset.

For COD datasets, we first present PR curves and F-measure curves for quantitative comparison. As shown in Figure 3, we observe that our model outperforms the other models in terms of the PR curve and $F_{\beta }$ curves. This is due to the feature fusion approach we use (see Section 3.4 for details).

Moreover, as listed in Table 1, our model obtains superior scores on four COD datasets under five public camouflaged map quality evaluation metrics. For instance, our model outperforms all the advanced models in five evaluation metrics for the CHAMELEON and COD10K datasets, achieving MAE of 0.026 and 0.034, respectively, which is 7.14% and 5.56% lower than FAPNet. Similarly, compared to FAPNet, the $F_{\beta }$ also improves by 2.06% and 1.66% on the CAMO and CHAMELEON datasets, respectively. Although our model’s $F_{\beta }^{w}$ scores rank second among the available models for the NC4K dataset, their scores only decrease by 0.26% . Furthermore, as shown in Table 2, our model achieves great results in categories within the COD10K dataset. For instance, in COD10K-Amphibian, compared with FAPNet, our model’s MAE decreases by 15.63% .

Overall, through Figure 3 and Table 1 and Table 2, the excellence and efficiency of our model, which has attained SOTA performance, are readily apparent.

#### 4.3.2. **Qualitative Comparison**

**Figure 4:**
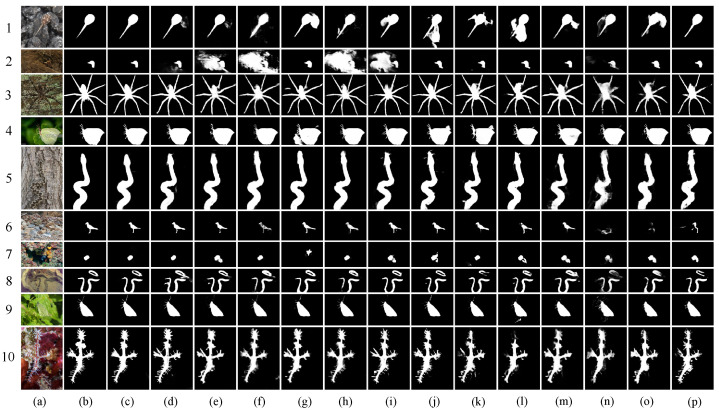
Visual comparison of our model and others on four COD testing datasets. (**a**) Input, (**b**) GT, (**c**) ours, (**d**) FAPNet [8], (**e**) SINet_V2 [1], (**f**) UGTR [53], (**g**) C^2^FNet [24], (**h**) MGL-R [52], (**i**) SLSR [2], (**j**) UJSC [25], (**k**) PFNet [3], (**l**) SINet [19], (**m**) PraNet [10], (**n**) ITSD [51], (**o**) MINet [50], (**p**) UCNet [49].

We carry out several visual contrast experiments and provide corresponding images to make a qualitative comparison for all models. As shown in Figure 4, our model’s detection results are more comparable to the GT, indicating that our results are more complete and precise than those of the other models. In general, our model has two major advantages:

(a) Object placement accuracy: In the first, second, seventh, and eighth rows of Figure 4, we can see that our model’s outcomes closely resemble the GT. In contrast, other deep-learning-based models, (e.g., (d) FAPNet [8], (e) SINet_V2 [1], (l) SINet [19], etc.), shown in Figure 4, find the object but mistake a portion of the background for the object in the process.

(b) Advantages of edge details for optimization: In the third, fourth, ninth, and tenth rows of Figure 4, our model is capable of precisely locating the object and properly identifying microscopic details. For other models, (e.g., (d) FAPNet [8], (e) SINet_V2 [1], (g) C^2^FNet [24], (l) SINet [19], etc.), although they may detect the object’s major portion, the object’s boundary is unclear, tailing is a serious occurrence, and the edge details are not readily apparent.

Based on the above comparisons, we can indisputably establish the efficacy and superiority of the FSANet that we present. When it comes to identifying camouflaged objects, whether they are inside the object or on its edge, our model performs better than the other models.

### 4.4. Ablation Studies

**Figure 5:**
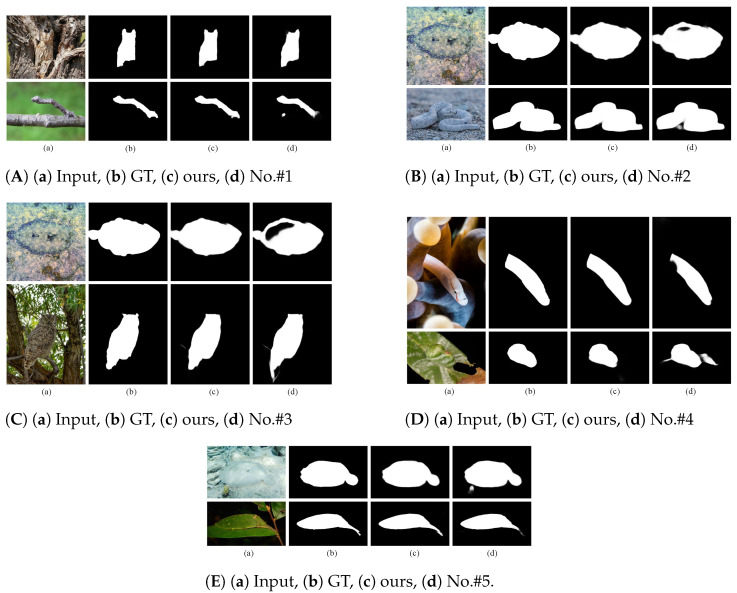
Qualitative comparisons of five experiments (**A**–**E**) No.#1–No.#5.

In this section, we conduct a thorough experiment on two COD datasets to demonstrate the efficacy of each model component. Table 3 displays the quantitative comparison; Figure 5A–E display the qualitative comparisons. We conduct experiments on the SDM, TEM, SJM, and HFAD modules to validate their effectiveness. The following are the details of the implementation of the experiment.

Table 3 demonstrates how various operations can be used to further enhance the model’s performance. When all the proposed modules are combined, our model performs the best, particularly when applied to the CAMO dataset, where our model performs better than any other stage. With relation to the model without many-to-many side-join multiplication, a one-to-many side-join multiplication (No.#5) is used, and the $S_{m}$ and MAE of ours in CAMO are improved by 3.67% and 5.56% , respectively. When we remove TEM from the CFC module and use Conv3 instead (No.#1), each of the two datasets’ metrics are noticeably worse; especially, $S_{m}$ and MAE show the most obvious decline. Experiment No.#3 verifies the effectiveness of HFAD; if we remove the HFAD, while the $F_{\beta }$ in the COD10K dataset improves marginally compared to ours, other indicators of our model significantly decrease; in particular, $E_{m}$ in the CAMO dataset declines 19.25% .

We also provide the prediction map of five ablation settings to visually demonstrate the effectiveness of our strategy. When we do not use TEM to enlarge the receptive field in the CFC module (No.#1), according to Figure 5A, the camouflaged objects can roughly be resolved, but the edge details are not smooth enough. As shown in Figure 5B,C, we introduce the SDM module and CFC module (No.#2, #3) to address the issue that the prediction map is void because the high-level semantic characteristics do not contain image spatial details and other information. Furthermore, we independently confirm the many-to-many and one-to-many side-join multiplication for the CFC module (No.#4, #5), as shown in Figure 5D,E; we improve the detection accuracy by re-adding the information that NCD* overlooked to the prediction feature using the many-to-many side-join multiplication technique that we devised.

It is demonstrated that our model fully complies with the anticipated design standards based on the qualitative analysis and quantitative analysis of the aforementioned ablation study.

### 4.5. Failure Cases and Analysis

**Figure 6:**
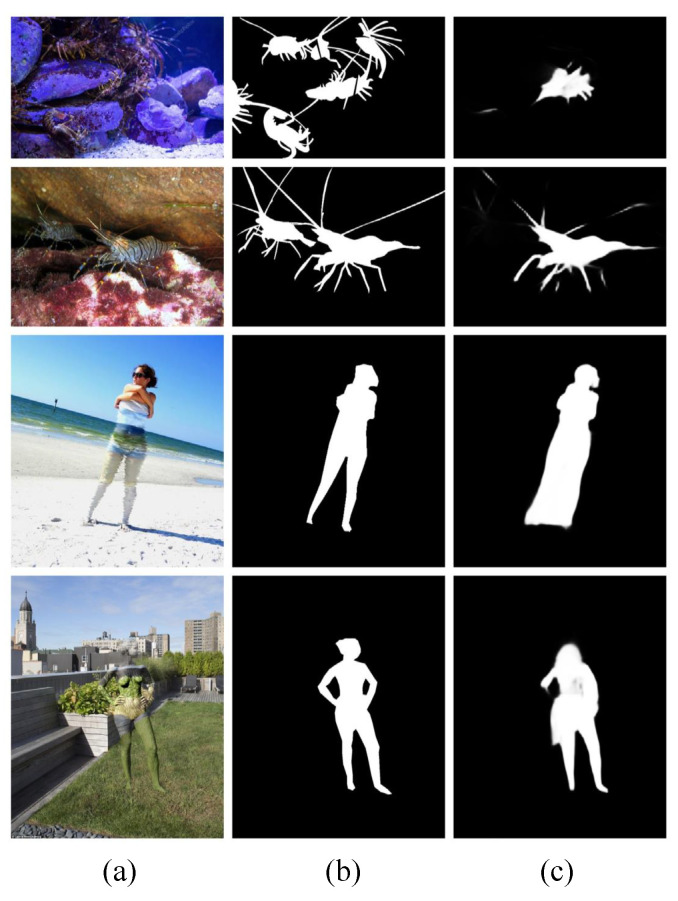
Failed cases: (**a**) input, (**b**) GT, (**c**) ours.

As shown in Table 4, we evaluate the inference speed of our model in comparison to other models. The findings demonstrate that, despite our model’s successful utilization of the SDM, CFC, and HFAD modules and achievement of the primary design goals, a significant amount of duplication still exists in our model. Thus, the model will be further developed from the efficiency standpoint. On the other hand, the first and second rows of Figure 6 depict certain failed scenarios where numerous camouflaged objects are present but only one can be detected by our model. This could be because the CFC module is being used, which focuses more on scenarios where there is only one camouflaged object and filters out other objects as background data. In our subsequent research, we will further explore methods for multi-object detection, such as instance segmentation [54]. Furthermore, the object’s edge processing is sloppy, and the background is wrongly identified as the foreground when using artificial camouflage, as demonstrated in the third and fourth rows. This could be as a result of the SDM module’s limited ability to effectively filter out interference data. The aforementioned results offer fresh perspectives for our upcoming model design.

## 5. Conclusions

In this paper, we propose a new model named features split and aggregation network (FSANet) to detect camouflaged objects, which can be divided into three modules to simulate the three-stage detection process of the human visual mechanism when viewing a camouflaged scene. To begin, we divide the backbone into two stages. The SDM module is used in the first stage to perform information interaction of first-level features to fully mine the spatial details (such as edge, texture, etc.) and fuse the features to create a cursory impression. In parallel, high-level semantic information from several sensory areas is mined by using the CFC module. Furthermore, we apply side-join multiplication in CFC to prevent detail distortion and reduce noise. Finally, we configure HFAD to completely fuse the effective information between the two stages to acquire more thorough detection results. Through in-depth experiments on four public camouflaged datasets, we observe that both quantitative and qualitative results verify the effectiveness of our methodology. These results prove the validity and superiority of our model. However, our model still has some limitations. When there are numerous camouflaged objects, our model can only detect one. Additionally, for artificially camouflaged objects, our model fails to perform fine-grained edge processing. The above results provide new directions for our upcoming model design. Furthermore, we aspire for our model to be adaptable across a broader range of applications, including but not limited to industrial defect detection and medical image segmentation and detection.

## Figures and Tables

**Table 1:** Quantitative comparison of different methods on four COD testing datasets, which contain S-measure ( $S_{m}$ ), weighted F-measure ( $F_{\beta }^{w}$ ), F-measure ( $F_{\beta }$ ), E-measure ( $E_{m}$ ), and mean absolute error ( MAE ). Here, “↑” (“↓”) means that the larger (smaller) the better. The best three results in each column are marked in red, green, and blue.

	CAMO Dataset					CHAMELEON Dataset					COD10K Dataset					NC4K Dataset				
	$S_{m}↑$	$F_{\beta }^{w}↑$	$F_{\beta }↑$	$E_{m}↑$	MAE↓	$S_{m}↑$	$F_{\beta }^{w}↑$	$F_{\beta }↑$	$E_{m}↑$	MAE↓	$S_{m}↑$	$F_{\beta }^{w}↑$	$F_{\beta }↑$	$E_{m}↑$	MAE↓	$S_{m}↑$	$F_{\beta }^{w}↑$	$F_{\beta }↑$	$E_{m}↑$	MAE↓
EGNet [44]	0.732	0.604	0.670	0.800	0.109	0.797	0.649	0.702	0.860	0.065	0.736	0.517	0.582	0.810	0.061	0.777	0.639	0.696	0.841	0.075
PoolNet [46]	0.730	0.575	0.643	0.747	0.105	0.845	0.691	0.749	0.864	0.054	0.740	0.506	0.576	0.777	0.056	0.785	0.635	0.699	0.814	0.073
F^3^Net [37]	0.711	0.564	0.616	0.741	0.109	0.848	0.744	0.770	0.894	0.047	0.739	0.544	0.593	0.795	0.051	0.780	0.656	0.705	0.824	0.070
SCRN [45]	0.779	0.643	0.705	0.797	0.090	0.876	0.741	0.787	0.889	0.042	0.789	0.575	0.651	0.817	0.047	0.830	0.698	0.757	0.854	0.059
CSNet [47]	0.771	0.642	0.705	0.795	0.092	0.856	0.718	0.766	0.869	0.047	0.778	0.569	0.635	0.810	0.047	0.750	0.603	0.655	0.773	0.088
SSAL [48]	0.644	0.493	0.579	0.721	0.126	0.757	0.639	0.702	0.849	0.071	0.668	0.454	0.527	0.768	0.066	0.699	0.561	0.644	0.780	0.093
UCNet [49]	0.739	0.640	0.700	0.787	0.094	0.880	0.817	0.836	0.930	0.036	0.776	0.633	0.681	0.857	0.042	0.811	0.729	0.775	0.871	0.055
MINet [50]	0.748	0.637	0.691	0.792	0.090	0.855	0.771	0.802	0.914	0.036	0.770	0.608	0.657	0.832	0.042	0.812	0.720	0.764	0.862	0.056
ITSD [51]	0.750	0.610	0.663	0.780	0.102	0.814	0.662	0.705	0.844	0.057	0.767	0.557	0.615	0.808	0.051	0.811	0.680	0.729	0.845	0.064
PraNet [10]	0.769	0.663	0.710	0.824	0.094	0.860	0.763	0.789	0.907	0.044	0.789	0.629	0.671	0.861	0.045	0.822	0.724	0.762	0.876	0.059
SINet [19]	0.745	0.644	0.702	0.804	0.092	0.872	0.806	0.827	0.936	0.034	0.776	0.631	0.679	0.864	0.043	0.808	0.723	0.769	0.871	0.058
PFNet [3]	0.782	0.695	0.746	0.842	0.085	0.882	0.810	0.828	0.931	0.033	0.800	0.660	0.701	0.877	0.040	0.829	0.745	0.784	0.888	0.053
UJSC [25]	0.800	0.728	0.772	0.859	0.073	0.891	0.833	0.847	0.945	0.030	0.809	0.684	0.721	0.884	0.035	0.842	0.771	0.806	0.898	0.047
SLSR [2]	0.787	0.696	0.744	0.838	0.080	0.890	0.822	0.841	0.935	0.030	0.804	0.673	0.715	0.880	0.037	0.840	0.766	0.804	0.895	0.048
MGL-R [52]	0.775	0.673	0.726	0.812	0.088	0.893	0.813	0.834	0.918	0.030	0.814	0.666	0.711	0.852	0.035	0.833	0.740	0.782	0.867	0.052
C^2^FNet [24]	0.796	0.719	0.762	0.854	0.080	0.888	0.828	0.844	0.935	0.032	0.813	0.686	0.723	0.890	0.036	0.838	0.762	0.795	0.897	0.049
UGTR [53]	0.784	0.684	0.736	0.822	0.086	0.887	0.794	0.820	0.910	0.031	0.817	0.666	0.711	0.853	0.036	0.839	0.747	0.787	0.875	0.052
SINet_V2 [1]	0.820	0.743	0.782	0.882	0.070	0.888	0.816	0.835	0.942	0.030	0.815	0.680	0.718	0.887	0.037	0.847	0.770	0.805	0.903	0.048
FAPNet [8]	0.815	0.734	0.776	0.865	0.076	0.893	0.825	0.842	0.940	0.028	0.822	0.694	0.731	0.888	0.036	0.851	0.775	0.810	0.899	0.047
**Ours**	**0.821**	**0.752**	**0.792**	**0.883**	**0.068**	**0.897**	**0.841**	**0.856**	**0.952**	**0.026**	**0.822**	**0.699**	**0.734**	**0.890**	**0.034**	**0.846**	**0.773**	**0.808**	**0.899**	**0.047**

**Table 2:** Quantitative comparison of different methods on four COD10K testing dataset categories, which contain S-measure ( $S_{m}$ ), weighted F-measure ( $F_{\beta }^{w}$ ), F-measure ( $F_{\beta }$ ), E-measure ( $E_{m}$ ), and mean absolute error ( MAE ). Here, “↑” (“↓”) means that the larger (smaller) the better. The best three results in each column are marked in red, green, and blue.

	COD10K-Amphibian					COD10K-Aquatic					COD10K-Flying					COD10K-Terrestrial				
	$S_{m}↑$	$F_{\beta }^{w}↑$	$F_{\beta }↑$	$E_{m}↑$	MAE↓	$S_{m}↑$	$F_{\beta }^{w}↑$	$F_{\beta }↑$	$E_{m}↑$	MAE↓	$S_{m}↑$	$F_{\beta }^{w}↑$	$F_{\beta }↑$	$E_{m}↑$	MAE↓	$S_{m}↑$	$F_{\beta }^{w}↑$	$F_{\beta }↑$	$E_{m}↑$	MAE↓
EGNet [44]	0.776	0.588	0.650	0.843	0.056	0.712	0.515	0.584	0.784	0.091	0.769	0.558	0.621	0.838	0.046	0.713	0.467	0.531	0.794	0.056
PoolNet [46]	0.781	0.584	0.644	0.823	0.050	0.737	0.534	0.607	0.782	0.078	0.767	0.539	0.610	0.797	0.045	0.707	0.441	0.508	0.745	0.054
F^3^Net [37]	0.808	0.657	0.700	0.846	0.039	0.728	0.554	0.611	0.788	0.076	0.760	0.571	0.618	0.818	0.040	0.712	0.490	0.538	0.770	0.048
SCRN [45]	0.839	0.665	0.729	0.867	0.041	0.780	0.600	0.674	0.818	0.064	0.817	0.608	0.683	0.840	0.036	0.758	0.509	0.588	0.784	0.048
CSNet [47]	0.828	0.649	0.711	0.857	0.041	0.768	0.587	0.656	0.808	0.067	0.809	0.610	0.676	0.838	0.036	0.744	0.501	0.566	0.776	0.047
SSAL [48]	0.729	0.560	0.637	0.817	0.057	0.632	0.428	0.509	0.737	0.101	0.702	0.504	0.576	0.795	0.050	0.647	0.405	0.471	0.756	0.060
UCNet [49]	0.827	0.717	0.756	0.897	0.034	0.767	0.649	0.703	0.843	0.060	0.806	0.675	0.718	0.886	0.030	0.742	0.566	0.617	0.830	0.042
MINet [50]	0.823	0.695	0.732	0.881	0.035	0.767	0.632	0.684	0.831	0.058	0.799	0.650	0.697	0.856	0.031	0.732	0.536	0.584	0.802	0.043
ITSD [51]	0.810	0.628	0.679	0.852	0.044	0.762	0.584	0.648	0.811	0.070	0.793	0.588	0.645	0.831	0.040	0.736	0.496	0.552	0.777	0.051
PraNet [10]	0.842	0.717	0.750	0.905	0.035	0.781	0.643	0.692	0.848	0.065	0.819	0.669	0.707	0.888	0.033	0.756	0.565	0.607	0.835	0.046
SINet [19]	0.820	0.714	0.756	0.891	0.034	0.766	0.643	0.698	0.854	0.063	0.803	0.663	0.707	0.887	0.031	0.749	0.577	0.625	0.845	0.042
PFNet [3]	0.848	0.740	0.775	0.911	0.031	0.793	0.675	0.722	0.868	0.055	0.824	0.691	0.729	0.903	0.030	0.773	0.606	0.647	0.855	0.040
UJSC [25]	0.841	0.742	0.769	0.905	0.031	0.805	0.705	0.747	0.879	0.049	0.836	0.719	0.752	0.906	0.026	0.778	0.624	0.664	0.863	0.037
SLSR [2]	0.845	0.751	0.783	0.906	0.030	0.803	0.694	0.740	0.875	0.052	0.830	0.707	0.745	0.906	0.026	0.772	0.611	0.655	0.855	0.038
MGL-R [52]	0.854	0.734	0.770	0.886	0.028	0.807	0.688	0.736	0.855	0.051	0.839	0.701	0.743	0.873	0.026	0.785	0.606	0.651	0.823	0.036
C^2^FNet [24]	0.849	0.752	0.779	0.899	0.030	0.807	0.700	0.741	0.882	0.052	0.840	0.724	0.759	0.914	0.026	0.783	0.627	0.664	0.872	0.037
UGTR [53]	0.857	0.738	0.774	0.896	0.029	0.810	0.686	0.734	0.855	0.050	0.843	0.699	0.744	0.873	0.026	0.789	0.606	0.653	0.823	0.036
SINet_V2 [1]	0.858	0.756	0.788	0.916	0.030	0.811	0.696	0.738	0.883	0.051	0.839	0.713	0.749	0.908	0.027	0.787	0.623	0.662	0.866	0.039
FAPNet [8]	0.854	0.752	0.783	0.914	0.032	0.821	0.717	0.757	0.887	0.049	0.845	0.725	0.760	0.906	0.025	0.795	0.639	0.678	0.868	0.037
**Ours**	**0.862**	**0.767**	**0.795**	**0.924**	**0.027**	**0.821**	**0.720**	**0.758**	**0.893**	**0.048**	**0.851**	**0.741**	**0.774**	**0.916**	**0.023**	**0.787**	**0.632**	**0.669**	**0.859**	**0.038**

**Table 3:** Ablation studies on two testing datasets. Here, m-m SJM represents many-to-many side-join multiplication (as shown by the red line in Figure 2); o-m SJM represents one-to-many side-join multiplication. Here, “↑” (“↓”) means that the larger (smaller) the better.

No.	SDM	CFC			Decoder		CAMO Dataset				COD10K Dataset			
	SDM	TEM	m-m SJM	o-m SJM	PD	HFAD	$S_{m}↑$	$F_{\beta }↑$	$E_{m}↑$	MAE↓	$S_{m}↑$	$F_{\beta }↑$	$E_{m}↑$	MAE↓
#1	✔		✔			✔	0.812	0.777	0.870	0.071	0.818	0.734	0.886	0.035
#2		✔	✔			✔	0.812	0.783	0.870	0.072	0.819	0.732	0.889	0.034
#3		✔	✔		✔		0.812	0.778	0.866	0.072	0.821	0.740	0.888	0.034
#4	✔	✔				✔	0.815	0.784	0.872	0.071	0.820	0.735	0.887	0.035
#5	✔	✔		✔		✔	0.818	0.784	0.874	0.072	0.821	0.730	0.885	0.035
**Ours**	✔	✔	✔			✔	**0.821**	**0.792**	**0.883**	**0.068**	**0.822**	**0.734**	**0.890**	**0.034**

**Table 4:** Comparisons of the number of parameters, FLOPs, and FPS corresponding to recent COD methods. All evaluations follow the inference settings in the corresponding papers.

Method	Ours	FAPNet [8]	SINet_V2 [1]	UGTR [53]	C^2^FNet [24]	MGL-R [52]	SINet [19]	SLSR [2]	UJSC [25]	PFNet [3]
Params.	66.550 M	29.524 M	26.976 M	48.868 M	28.411 M	63.595 M	48.947 M	50.935 M	217.982 M	46.498 M
FLOPs	40.733 G	59.101 G	24.481 G	1.007 T	26.167 G	553.939 G	38.757 G	66.625 G	112.341 G	53.222 G
FPS	29.417	28.476	38.948	15.446	36.941	12.793	34.083	32.547	18.246	29.175

## Data Availability

The data used to support the findings of this study are available from the corresponding author upon request.
